# Spatially resolved immune niches in thyroid cancer: from hot–cold–excluded ecosystems to precision immunotherapy

**DOI:** 10.3389/fimmu.2026.1863184

**Published:** 2026-07-01

**Authors:** Guizhang Hou, Tianshu Gao

**Affiliations:** 1 The First Clinical College, Liaoning University of Traditional Chinese Medicine, Shenyang, China; 2 Department of Endocrinology, Affiliated Hospital of Liaoning University of Traditional Chinese Medicine, Shenyang, China

**Keywords:** immunotherapy, spatial transcriptomics, tertiary lymphoid structures, thyroid cancer, tumor immune microenvironment

## Abstract

Although the overall prognosis of most thyroid cancers is relatively good, the benefits of immunotherapy in advanced, dedifferentiated, and some special subtypes still show significant heterogeneity. The existing evaluation frameworks based on PD-L1, tumor mutational burden, or conventional transcriptomic signals are insufficient to explain the complex and variable immune response patterns among different patients and within the same tumor. In recent years, single-cell sequencing, spatial transcriptomics, and related spatial multi-omics studies have shown that the immune microenvironment of thyroid cancer is not a homogeneous background but is composed of multiple local ecological niches with clear spatial organizational characteristics. These ecological niches have significant differences in cell composition, functional state, and interaction mode. The current evidence suggests that the regions rich in B cells and tertiary lymphoid structures in papillary thyroid carcinoma are often associated with relatively indolent clinical behaviors; undifferentiated thyroid carcinoma more frequently presents as an inhibitory spatial pattern characterized by macrophages, cancer-associated fibroblasts, and immune exclusion boundaries; and the neural-immune crosstalk in medullary thyroid carcinoma further indicates that some “cold” immune phenotypes may be actively shaped by neuroendocrine signals. From the perspective of spatial immune niches, this article re-examines the biological basis and translational significance of hot, cold, and excluded immune patterns in thyroid cancer, and discusses their potential implications for immune classification, biopsy strategies, and the optimization of precise immunotherapy.

## 1 Introduction

Thyroid cancer is not the most intensively studied type of solid tumor in terms of immunotherapy. However, as the clinical challenges of advanced, dedifferentiated, and treatment-resistant cases have become increasingly prominent, the value of immunotherapy for this type of cancer is being re-evaluated. In recent years, immune checkpoint inhibitors and their combined strategies with targeted therapy have shown certain activity in some aggressive thyroid cancers, especially in undifferentiated thyroid cancers and other highly malignant subtypes (1, 2). This suggests that immune regulation is not an irrelevant variable. Nevertheless, the existing efficacy remains unstable, and significant differences can be observed between different patients and even different regions of the same tumor. This indicates that relying solely on PD-L1 expression, tumor mutation burden, or bulk transcriptome signals is insufficient to explain the true and complex immunoreactivity of thyroid cancer (1, 2).

Meanwhile, single-cell and spatial omics studies are transforming our understanding of the immune microenvironment of thyroid cancer. Increasing evidence suggests that thyroid cancer is not simply an “immunologically cold” or “immunologically hot” tumor, but rather more like a disease spectrum composed of multiple local immune niches with clear tissue coordinates (3–5). The remodeling of the immune-stromal structure during dedifferentiation (4, 5), the emergence of B cells and the regions related to tertiary lymphoid structures in papillary thyroid cancer (6), as well as the discovery of neural-immune crosstalk in medullary thyroid cancer (7), all indicate that the spatially organized local interfaces may be more capable of determining tumor behavior and treatment sensitivity than averaged signals. Based on this, this article discusses the formation basis of the hot, cold and excluded immune patterns in thyroid cancer and their potential significance for immune typing and precise treatment from the perspective of spatial immune niches.

## 2 Why thyroid cancer needs a spatial immunology perspective

Thyroid cancer has not been a typical research subject in the field of immunotherapy for a long time. On one hand, most differentiated cases have a good prognosis after surgery, radioactive iodine treatment, and thyroid stimulating hormone suppression therapy. On the other hand, the truly challenging cases are often the few that are progressive, de-differentiated, or resistant to treatment. Therefore, the immunological research on thyroid cancer in the past has mostly focused on “whether there is immune infiltration” or “whether a certain immune checkpoint is expressed”, rather than delving deeper into how these immune components are distributed in the tissue, how they interact with each other, and why they exhibit distinct functional consequences in different regions (1, 2).

However, this kind of understanding based on average signals is becoming increasingly insufficient. Recent studies in single-cell and spatial omics have repeatedly indicated that even within the same lesion, thyroid cancer may coexist with multiple local states such as immune activation, immune exclusion, and immune suppression (3–5, 8). What determines the tumor behavior and treatment response is often not whether a certain type of cell “exists”, but whether these cells can enter the tumor parenchyma, whether they are confined to the peritumoral stroma, and what kind of local interfaces they form with tumor cells, fibroblasts, and myeloid cells (4, 5, 8).

In this sense, thyroid cancer cannot simply be classified as either “immunologically cold tumor” or “immunologically hot tumor”. A more reasonable framework might be to view it as a disease spectrum composed of multiple spatially organized local immune niches: some areas allow lymphocytes to gather and maintain a more favorable immune dialogue, while others are dominated by fibroblasts or myeloid cells, forming physical and functional dual barriers (3, 5, 8). For immunotherapy, these differences are not descriptive details but may very well be the key to explaining the unstable efficacy, inconsistent sample results, and the increased need for combined treatments. Therefore, spatial immunology should be viewed not as a simple technical supplement, but as a complementary framework for interpreting immune heterogeneity in thyroid cancer. Importantly, most current evidence remains observational and largely cross-sectional (3–5, 8). Spatial co-localization, niche enrichment, or cell–cell proximity does not by itself prove that a given immune niche causally drives progression or therapeutic resistance. A more cautious interpretation is that spatial immune niches provide testable biological hypotheses and clinically relevant sampling contexts, rather than fully validated causal classifications. This distinction is essential when translating hot, cold, and excluded patterns into prognostic or therapeutic decision-making.

## 3 From dissociated cells to intact tissue: technologies redefining the thyroid cancer immune landscape

Single-cell sequencing has fundamentally changed the way we observe the immune microenvironment of thyroid cancer. Early scRNA-seq studies have shown that papillary thyroid cancer is not composed of a single tumor cell population and uniform immune infiltration, but rather includes different differentiated tumor cells, functionally stratified T cell populations, as well as myeloid and stromal components with pro-invasive or immunosuppressive potential (3, 4). Subsequently, integrated analyses targeting the dedifferentiation process further suggest that when thyroid cancer evolves from a differentiated type to an undifferentiated type, not only does the tumor cell state change occur, but also the synchronous formation of an immunosuppressive microenvironment takes place (4, 8).

However, the limitations of dissociated-cell data are becoming increasingly evident. After tissue digestion, the cell adjacency relationships, infiltration boundaries, and tumor-stroma interfaces are partially smoothed out, and these pieces of information may precisely determine whether immune cells can enter the tumor parenchyma, whether they are confined to the periphery, and which cell combinations dominate the local immunosuppression. In the past two years, spatial transcriptomic studies have begun to reintroduce these missing pieces of information into the tissue coordinate system: on the one hand, spatial heterogeneity related to prognosis has been observed in PTC (9); on the other hand, spatial analysis across differentiation states further reveals that as dedifferentiation deepens, changes such as the expansion of myeloid cells and fibroblasts, the reduction of NK/endothelial components, and the strengthening of the immunosuppressive interface have clear spatial organizational characteristics (5, 8).

At the same time, multiple immunostaining and related spatial protein analysis are becoming important bridges connecting omics-based discoveries with pathological validation (10). As a result, the focus of thyroid cancer immunology research is shifting from “which cells are there” to “where are these cells organized, how they are organized, and how this organizational pattern shapes the treatment response (5, 9, 10).” These approaches restore tissue context, but their outputs should still be paired with functional, longitudinal, or outcome-linked validation before being interpreted as causal mechanisms (11).

## 4 Spatial immune niches across thyroid cancer subtypes

### 4.1 Papillary thyroid cancer

Among the various subtypes of thyroid cancer, PTC best demonstrates that the “spatial immune niche” is not an additional description but an important level for understanding the behavior of the tumor. Early single-cell studies have shown that the immune microenvironment of PTC is not uniform: tumor cells with different differentiation states can form distinct local combinations with T cells, myeloid cells, and stromal components (3). Recent studies have further indicated that what truly deserves attention within PTC is not merely “the extent of immune infiltration”, but rather which areas allow for the formation of a relatively favorable local immune dialogue, and which areas gradually shift to a restricted or imbalanced state.

The most prominent clues currently come from the regions related to B cells and tertiary lymphoid structures. The 2025 single-cell study revealed that in indolent PTC, tumor-infiltrating B cells, especially germinal center B cells, are more abundant and more likely to form TLS within the tumor; these local structures not only suggest a more active humoral immunity involvement, but are also associated with a milder clinical behavior and better disease outcomes (6, 12). Mechanistically, TLS should not be interpreted merely as lymphocyte aggregates. In cancer tissues, mature TLS usually contain organized B-cell follicles, T-cell zones, dendritic-cell networks, and high endothelial venule-like structures, supported by chemokine programs such as CXCL13, CCL19, and CCL21. These structures may facilitate local antigen presentation, B-cell maturation, antibody production, and T-cell priming (13, 14), thereby converting a diffuse immune infiltrate into a more organized anti-tumor immune niche. In PTC, the enrichment of germinal-center-like B cells and TLS-associated regions therefore provides a plausible explanation for why some tumors show indolent behavior despite variable overall immune infiltration. However, whether TLS directly restrain PTC progression or simply mark a less aggressive tumor state remains unresolved and requires spatially matched functional validation.

**Figure 1:**
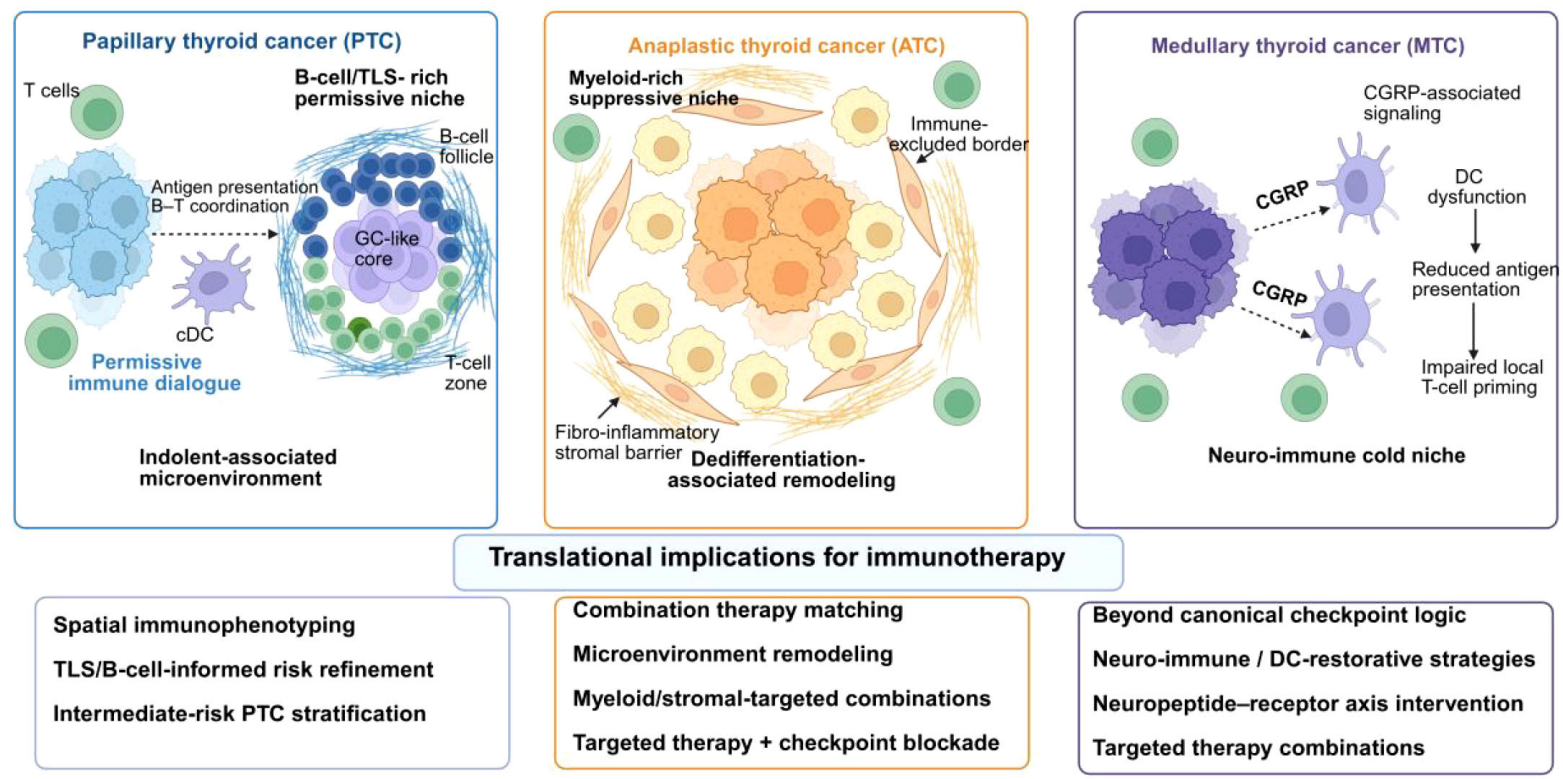
Subtype-specific spatial immune niches in thyroid cancer and their translational implications for immunotherapy. This conceptual figure summarizes representative spatial immune architectures across papillary thyroid cancer (PTC), anaplastic thyroid cancer (ATC), and medullary thyroid cancer (MTC). PTC is illustrated as a B-cell/TLS-rich permissive niche, including a B-cell follicle, GC-like core, T-cell zone, and cDC-associated antigen presentation/B–T-cell coordination, which together reflect a more organized local immune dialogue associated with relatively indolent behavior. ATC is depicted as a suppressive and immune-excluded ecosystem characterized by myeloid enrichment, fibro-inflammatory stromal barriers, excluded T cells, and dedifferentiation-associated remodeling. MTC is shown as a neuro-immune cold niche in which CGRP-positive tumor cells are linked to dendritic-cell dysfunction, reduced antigen presentation, and impaired local T-cell priming. The lower panel summarizes corresponding translational implications, including spatial immunophenotyping and intermediate-risk PTC stratification, combination therapy matching and microenvironment remodeling in ATC, and neuro-immune or dendritic-cell-restorative strategies in MTC.

At the same time, the spatial immune landscape of PTC does not always develop in a favorable direction. Spatial transcriptomic studies have revealed that the cell heterogeneity related to prognosis has a clear distribution in tissues (9), and recent work has also suggested that certain tumor regions can be driven by inflammatory macrophages, specific tumor cell populations, and local stromal remodeling, forming a microenvironment more prone to recurrence and progression (15). In other words, the key issue in PTC is not whether immunity is present, but how protective B-cell/TLS-rich niches are dynamically balanced against the myeloid–tumor–stroma interface. This distinction may be particularly relevant for patients who fall into clinically intermediate categories, in whom conventional histopathological features identify recurrence risk but do not fully capture the local immune architecture that may influence indolent versus progressive behavior. Therefore, PTC is more suitable to be understood as a disease composed of multiple local immune states, which also provides a more detailed basis for its risk stratification and future immune classification (3, 6, 9, 15) (**Figure 1**).

### 4.2 Anaplastic thyroid cancer

If PTC better reflects the coexistence and balance among local immune niches, then ATC more prominently exposes how the spatial immune structure undergoes inhibitory remodeling during dedifferentiation. Single-cell studies have shown that the immune landscape of ATC is not simply immune-poor, but is more often characterized by dysfunctional immune accumulation and stromal reinforcement, among them, the enrichment of exhausted T cells, myeloid suppressor cells, and migratory fibroblasts is particularly prominent (16, 17). More recent studies have further suggested that the internal composition of ATC is not completely homogeneous. The immune-fibrosis-enriched subpopulations often have higher expression of immune checkpoints, higher EMT activity, and more significant expansion of myeloid/fibroblastic cells, suggesting that the local spatial pattern itself may be an important component of invasiveness (16, 17).

Observations at the spatial level further reinforce this view. Spatial transcriptomic studies across differentiation states have shown that as thyroid cancer progresses from differentiated type to undifferentiated type, there are changes in the tumor area such as expansion of myeloid cells and fibroblasts, reduction of NK and endothelial components, and enhancement of immunosuppressive interfaces with clear tissue-coordinate features (5, 8). This means that the key issue in ATC is not simply “the presence or absence of immunity”, but whether immune cells are trapped in ineffective or tumor-promoting local circuits, and whether the tumor–stromal boundary constitutes both a physical and functional barrier.

However, these observations should be interpreted as suppressive spatial associations rather than definitive proof of causal hierarchy. Whether the myeloid–fibroblast exclusion architecture is a primary driver of immune resistance, a downstream consequence of dedifferentiation, or part of a reciprocal feed-forward loop remains unresolved. At a finer mechanistic level, recent studies suggest that macrophage-associated signals, including SPP1-related programs, SIGLEC15 upregulation, and M2 macrophage–linked CPA4 induction, may contribute to fibro-inflammatory remodeling and ineffective antitumor immunity in ATC (18, 19). Nevertheless, their spatial distribution, temporal sequence during dedifferentiation, and functional relationship with T-cell exclusion still require further validation.

This framework also helps to explain why the treatment of ATC relies more on combined strategies. A prospective non-randomized study in 2024 demonstrated that targeted therapy based on driver gene matching combined with PD-L1 inhibitors could improve outcomes in some patients, suggesting that the immunotherapy value of ATC is more likely to be based on the dual foundation of “rapid tumor control + microenvironment modification” rather than a single checkpoint blockade (2). Viewed from the perspective of spatial immune niches, ATC therefore provides a biologically plausible, but still clinically unvalidated, rationale for myeloid reprogramming, stromal barrier modulation, and mechanism-based combinatorial immunotherapy (2, 16, 18). At present, these spatial features are best considered mechanistic hypotheses and candidate stratification variables for future biomarker-guided studies rather than ready-to-use predictive biomarkers.

### 4.3 Medullary thyroid cancer

Compared with PTC and ATC, the spatial immune ecosystem of MTC is more appropriately understood as a “cold niche” with neuroendocrine characteristics. The core issue is not merely the insufficient number of immune cells, but rather the inhibition of the local immune activation process itself. A single-cell study conducted in 2024 comparing MTC with PTC found that the overall immune infiltration in MTC was lower, particularly manifested as abnormal development of dendritic cells and impaired function of tumor-infiltrating T cells; more importantly, this inhibition was not an isolated phenomenon but was closely related to the high expression of CGRP in tumor cells (7). This study suggests that CGRP can affect the differentiation of dendritic cells through its receptor, accompanied by the activation of cAMP-related pathways and the upregulation of KLF2, thereby weakening antigen presentation and co-stimulatory ability, and further restricting local T cell activation (7).

This observation places MTC somewhat apart from the conventional notion of an immunologically cold tumor (20, 21). For MTC, the local immunological low reactivity is more likely the result of active shaping by neuro-immune crosstalk rather than a simple lack of immune recognition. Therefore, the spatial ecological niche of MTC should not merely be described as “immune-poor”, but rather be regarded as a neuro-immune suppressive niche: the neuro-peptide signals secreted by tumor cells are coupled with antigen presentation disorders, low effector states of T cells, and insufficient local immune activation, jointly constituting a functionally “cold” microenvironment (7, 20, 22).

From a translational perspective, this framework suggests that the immunotherapy obstacles of MTC may be partially located outside the classical checkpoint axis (20, 22, 23). Instead of simply classifying MTC as an immunotherapy-insensitive tumor, it may be more informative to examine the neuropeptide–receptor axis, dendritic-cell dysfunction, and targeted therapy combinations within the same framework (20, 22, 23). Nevertheless, this interpretation should remain cautious. First, the current CGRP-related evidence is still mainly mechanistic and preclinical, and CGRP expression has not yet been established as a clinically validated predictive biomarker in MTC (7, 20). Second, CGRP-high states may reflect neuroendocrine lineage identity as well as active immune suppression, making it difficult to separate marker function from driver function (7, 21). Third, whether CGRP-mediated immunosuppression is stable across primary and metastatic lesions, or changes after RET-directed or multikinase inhibitor therapy, remains unclear (20). Therefore, CGRP should be presented as a hypothesis-generating neuro-immune axis rather than an immediately actionable immunotherapy target (7, 20, 22, 23).

## 5 Translational implications for immunotherapy

Translationally, the significance of the spatial immune ecological niche lies in that it provides a more detailed immune classification framework for thyroid cancer than a single PD-L1 or bulk signal. For PTC, the enrichment areas of B cells and TLS suggest that local immune constraints still exist. These characteristics are expected to serve as supplementary clues for identifying a more favorable immune background in the future (6, 24); conversely, in ATC, the suppressive or immune-excluded architectures dominated by myeloid cells and fibroblasts suggest that checkpoint expression alone may not be sufficient to predict benefit, and the local microenvironment status also needs to be taken into account for judgment (2, 5, 25).

Spatial immune profiling may have its greatest near-term value in intermediate-risk PTC, where conventional clinicopathological variables can identify recurrence risk but may not fully explain why some tumors remain indolent whereas others progress (26). In this setting, TLS- or B-cell-enriched permissive niches could serve as exploratory markers of a more organized local anti-tumor response, whereas macrophage-rich, stromal-remodeled, or immune-excluded regions may indicate a higher-risk local interface (6, 9, 12, 15). Such information should not replace established risk stratification systems (26), but it may refine tissue-level interpretation, guide sampling of heterogeneous lesions, and help identify patients who require closer surveillance or additional molecular-pathological evaluation.

Secondly, the spatial perspective has changed our understanding of biopsy and biomarker sampling. If there are permissive, excluded and suppressive niches within the same tumor, the results obtained from a single sampling point, such as PD-L1, immune cell infiltration or transcriptome, may be closer to a snapshot of a specific local interface rather than the true immune state of the entire lesion. For tumors like thyroid cancer, where spatial heterogeneity has been increasingly confirmed, a more reasonable strategy in the future may not be simply to increase the number of detection indicators, but to enhance the weight of sampling location, tissue structure and spatial pathological information in the interpretation (5, 9, 24).

More importantly, the spatial immune niche provides a more biologically grounded matching concept for combined therapy. The prospective non-randomized study of ATC demonstrated that targeted therapy based on driver gene matching combined with PD-L1 inhibitors could improve the outcomes of some patients, which is consistent with the understanding that a dual strategy of “rapid tumor control + microenvironment modification” is needed (2); while in MTC, the CGRP-related neuro-immune-suppressive program suggests that what is more worthy of exploration in the future is not simply following the conventional ICI logic, but integrating dendritic cell function restoration, neuropeptide-receptor axis intervention, and molecular targeted therapy into a translational framework (7, 20, 23). Therefore, the true value of spatial immune typing may not lie in adding a new technical label, but in helping immunotherapy move from empirical attempts to more mechanism-oriented treatment matching (24, 25).

## 6 Challenges and future directions

### 6.1 Technical and analytical limitations

Although spatial omics is significantly enhancing our understanding of the immune ecosystem of thyroid cancer, its clinical translation still faces several practical bottlenecks. These limitations should be stated before spatial immune signatures are discussed as clinical tools. Thyroid cancer, especially preoperative fine-needle aspiration specimens, often have limited tissue quantities, and the current spatial transcriptomic platforms still have trade-offs in resolution, gene detection depth, and tissue preservation conditions (24, 25); this means that although some local interfaces have biological significance, they may not be stably captured in routine clinical samples. For relatively rare subtypes such as ATC and MTC, the sample sizes are smaller and the differences between centers are greater, which further limits the external validation of spatial features (5, 8, 24).

### 6.2 Clinical implementation barriers

The second issue lies in the insufficient interpretation of results and standardization. Currently, most spatial studies on thyroid cancer come from small sample cohorts, and there are still differences among different platforms, analysis procedures, and cell annotation strategies (24, 25), which leads to concepts such as “immune exclusion boundary”, “myeloid enrichment area”, or “protective TLS niche” being attractive yet not yet forming a unified standard that can be directly transferred. Moreover, although the immune-stromal remodeling related to dedifferentiation has been observed in multiple studies, whether these spatial features are stable classifications or change dynamically with treatment and disease progression still needs larger sample sizes and longitudinal studies to clarify (5, 8). At present, there are no universally accepted cutoffs for defining a TLS-rich niche, an immune-excluded boundary, or a myeloid-dominant suppressive region in thyroid cancer.

### 6.3 From spatial atlases to surrogate panels

A more realistic direction is not to apply high-throughput spatial omics to all patients. Instead, research-level spatial atlases should first be used to define recurrent ecological patterns, which can then be converted into simplified and reproducible pathological or protein-based surrogate panels (10, 24, 27). A practical next step may be to derive simplified multiplex immunofluorescence surrogate panels from research-grade spatial atlases, such as a Pan-CK/CD8/CD68 (or CD163)/α-SMA (or FAP)/PD-L1 panel for suppressive or immune-excluded ATC-like niches, or a Pan-CK/CD20/CD3 (or CD8)/DC-LAMP (or PNAd)/PD-L1 panel for TLS- or B-cell-associated permissive niches in PTC. These should be viewed as exploratory translational candidates rather than ready-to-use clinical assays (10, 27, 28). For thyroid cancer, the most realistic next step is not simply to increase technical complexity, but to establish spatial immune signature systems that preserve biological resolution while remaining feasible for classification, sample interpretation, and prospective treatment matching (24, 25, 29).
